# The correlation of BTLA rs1982809 polymorphism with cancer susceptibility: A meta-analysis of 8634 participators

**DOI:** 10.1097/MD.0000000000029610

**Published:** 2022-08-05

**Authors:** Jian Chen, Jun Wang, Ruihao Liu, Haiwei Xiong, Yingying Liu, Mingzhi Zha, Qiang Li, Xuan Liu, Mingjun Shang, Yingliang Li

**Affiliations:** a General Surgery Department, First Affiliated Hospital of Nanchang University, Nanchang, Jiangxi; b General Surgery Department, Jing’an people’s Hospital, Yichun, Jiangxi; c Anesthesiology Department, First Affiliated Hospital of Nanchang University, Nanchang, Jiangxi.

**Keywords:** BTLA, cancer susceptibility, meta-analysis, rs1982809, single-nucleotide polymorphisms

## Abstract

**Background:**

The connection between B and T lymphocyte attenuator rs1982809 polymorphism and cancer risk has been investigated by several studies and yielded different results. Therefore, we adopted the meta-analysis method to assess the association of rs1982809 polymorphism with the susceptibility of cancers synthetically.

**Methods:**

Eligible publications were gathered by retrieving PubMed, Web of Science, Embase, Wan Fang, and China National Knowledge Infrastructure. We utilized odds ratio (OR) and 95% confidence intervals (95% CI) to assess correlation intensity and performed subgroup analyses, sensitivity analyses, and publication bias assessments.

**Results:**

Six researches that encompassed 3678 cases and 4866 controls were incorporated into our meta-analysis. The rs1982809 polymorphism was proved to be connected with cancer risk by the meta-analysis in the additive model (G vs A: OR *=* 1.11, 95% CI *=* 1.04–1.19, *P*_heterogeneity_ *=* .096). Subgroup analyses revealed that this SNP is regarded as a susceptible factor for cancers in the dominant, heterozygous, and additive model (AG + GG vs AA: OR *=* 1.46, 95% CI *=* 1.19–1.80, *P*_heterogeneity_ *=* .592; AG vs AA: OR *=* 1.47, 95% CI *=* 1.19–1.82, *P*_heterogeneity_ *=* .536; G vs A: OR *=* 1.32, 95% CI *=* 1.12–1.55, *P*_heterogeneity_ *=* .745) in Caucasians; And this SNP may increase the susceptibility to lung cancer (GG vs AG+AA: OR *=* 1.20, CI *=* 1.01–1.44, *P*_heterogeneity_ *=* .854; G vs A: OR *=* 1.17, CI *=* 1.02–1.33, *P*_heterogeneity_ *=* .232).

**Conclusion:**

The paper concludes that B and T lymphocyte attenuator rs1982809 polymorphism may contribute to cancers, especially in Caucasians, and it may associate with lung cancer.

## 1. Introduction

As the primary cause of morbidity and mortality worldwide and in every region of the world, cancers place a tremendous burden on the global medical systems, regardless of the level of human development.^[1]^ So many causes, including diet, lifestyle, heredity, and environmental factors, contribute to tumors. Hereditary factors probably play a crucial role in the genesis and progression of cancers among them. Moreover, molecular epidemiological studies have shown that genetic factors, such as single nucleotide polymorphisms (SNPs), might also be associated with the pathogenesis of cancers.^[2–4]^

Many studies have revealed that the immunity system has played a significant role in preventing tumorigenesis and tumor progression during the past decade.^[5]^ Immunotherapy has ushered cancer treatment into a new era,^[6]^ programmed death 1 (PD-1) and cytotoxic T lymphocyte-associated antigen 4 (CTLA-4) inhibitors are particularly prominent in this field. As the third inhibitory receptor on T lymphocytes, BTLA is similar to PD-1 and CTLA-4,^[7]^ dampening immune responses. BTLA molecule plays a significant role in sustaining immunological self-tolerance and preventing autoimmunity,^[8,9]^ but its high level of expression can reduce the body’s immunity and cause people to develop various diseases, even cancers. It is reported that the overexpression of BTLA has been observed in a variety of tumors.^[10]^

The BTLA gene, composed of 5 exons, is located on the q13.2 region of chromosome 3^[11]^ and expressed on B and T lymphocytes, antigen-presenting cells, and natural killer cells.^[7]^ An extracellular domain, a transmembrane region, and a cytoplasmic region are contained in the BTLA.^[12]^ Herpesvirus entry mediator (HVEM), the ligand of BTLA, belongs to TNF family members.^[13]^ When BTLA bound its ligand (HVEM), the tyrosine of the cytoplasmic region in the BTLA gene will be phosphorylated by recruiting Src homology phosphatase-1 and Src homology phosphatase-2 to significantly lower the serum level of IL-1, IL-10, and IFN-γ, and some studies have utilized the mouse models to identify the role of BTLA/HVEM pathway.^[14]^ As a CD28/B7 superfamily member, BTLA has a similar framework and functions to PD-1 and CTLA-4, which delivers prohibitive signals to lymphocyte cells. Therefore, this negative effect will be magnified when the organism is pathological or under some genetic factors. Some SNPs in BTLA may cause its overexpression to increase the risk of some diseases, and this has been shown in some studies. Two studies indicated that BTLA gene polymorphism contributes to the occurrence and progression of rheumatoid arthritis.^[15,16]^ The research of Fu et al. suggests BTLA gene polymorphisms might be correlated with susceptibility and prognosis of sporadic breast cancer in Chinese women.^[17]^ During the past few years, increasing studies have found SNPs in BTLA are a risk factor for tumorigenesis and tumor progression, especially rs1982809. Lidia Karabon and Anna Partyka found that SNP in the BTLA rs1982809 was an increased risk factor for chronic lymphocytic leukemia (CLL) and renal cell carcinoma (RCC) in the Polish people.^[18,19]^ Tang et al. reported that rs1982809 polymorphism was proved to be correlated with Esophagogastric junction adenocarcinoma (EGJA) in smoking patients.^[20]^ This SNP is not associated with Esophageal squamous cell carcinoma (ESCC) in Chinese in the study of Cao et al.,^[14]^ and the latest study in Tunisia showed that BTLA rs1982809 might cause lung cancer (LC)^[21]^. However, Wang et al. found that rs1982809 polymorphism reduced the risk of LC.^[22]^ Based on the above, we conducted a meta-analysis to comprehensively assess the relationship between BTLA rs1982809 polymorphism and tumor susceptibility.

## 2. Methods

All data of this study were rooted in published literature and did not involve patients directly. Hence, we do not need the approval and informed consent of the ethics committee.

The protocol for this systematic review was registered on INPLASY (202130023) and is available in full on inplasy.com (https://doi.org/10.37766/inplasy2021.3.0023).

### 2.1. Retrieval strategy

Eligible researches incorporated in this meta-analysis were screened and identified in 5 online literature data banks (PubMed, Web of Science, Embase, Wan Fang, and CNKI) by 2 independent investigators. The keywords we utilized were: “B and T lymphocyte attenuator or BTLA or LOC112268446 or rs1982809 or rs386551325 or rs60386396” and “neoplasm or tumor or cancer or carcinoma” and “polymorphism or SNP or allele or variation”. There are no language restrictions on the search for articles. The search deadline is January 2021.

### 2.2. Identify available literature

Researches were filtrated based on the inclusion criteria:(a) the studies investigated the connection between BTLA rs1982809 polymorphism and tumor risks; (b) the studies were case-control studies, tumor patients were included in the case group and healthy people were included in the control group; (c) the studies provided detailed genotype frequencies. Exclusion criteria:(a) non-human trials; (b) duplicate articles. Inconsistencies between the 2 researchers were discussed with a third till a consensus was reached.

### 2.3. Data extraction

The data, including first author, publication year, country, ethnicity, cancer types, control source, genotype frequencies of the case and controls, and a *P*-value of the HWE test for the control groups, were extracted by 2 independent researchers from each eligible publication. Each item was researched by consensus by 2 reviewers.

### 2.4. Quality assessment

Both researchers assessed the quality of the included experiments based on the Newcastle–Ottawa Scale (NOS). The case-control trials were scored on 3 dimensions: selection, comparability, and exposure, with a total score of 9. A score of 5–9 was considered high quality, while a score of 0–4 was considered low quality. In case of disagreement between 2 investigators, discussion with a third party was required until an agreement was reached between the 3 parties.

### 2.5. Statistical analysis

All statistical analyses included in our study were enforced by the STATA 12.0. Pooled ORs and 95% CI were utilized to assess the relation between BTLA rs1982809 polymorphism and tumor susceptibility in the dominant, recessive, homozygous, and heterozygous additive models. We used the χ² test to measure the HWE for each study; the standard for which studies conformed to HWE is *P* > .05. ORs were merged via the random or fixed-effects model, and the selection of models depends on the heterogeneity of studies. We used the *Q* and *I*² tests to evaluate the heterogeneity of genetic models. The random-effects model was applied when heterogeneity exists in available studies (*P* < .05, *I*² > 50%); oppositely, the fixed-effects model was utilized. Sensitivity analysis was applied to evaluate the stability of the results via the leave-one-out method. Begg’s test was utilized for evaluating the publication bias of 5 genetic models; statistical significance was hypothesized at *P* < .05. Subgroup analysis based on the ethnicity, cancer type, and control source was conducted.

## 3. Results

### 3.1. Characteristics of eligible studies

**Figure 1.**
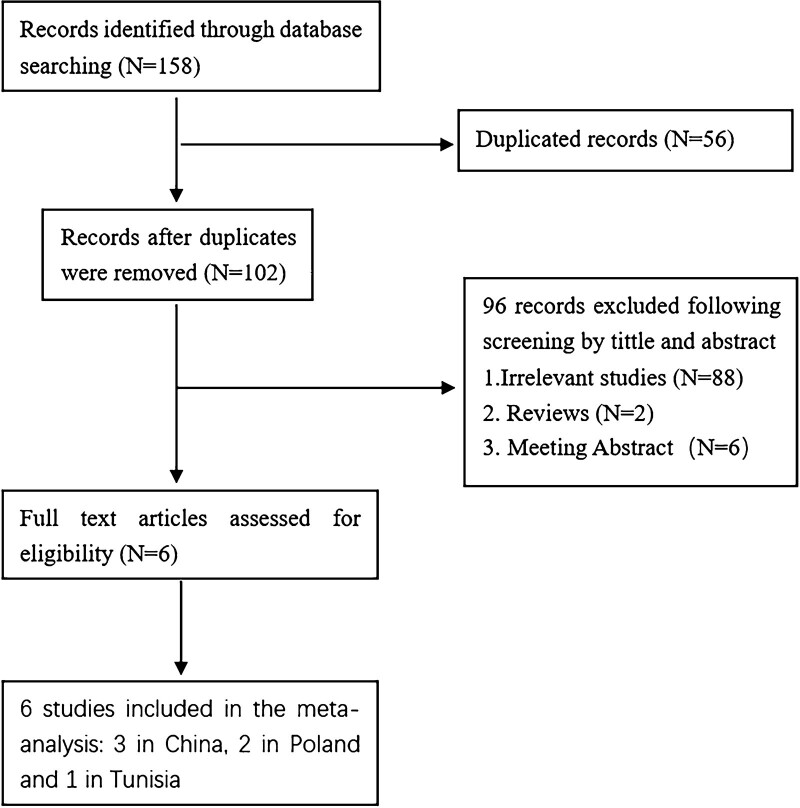
Flow chart of the screening process of eligible studies.

158 records were retrieved based on the primal search strategy. A total of 56 duplicated records were excluded, 96 articles were excluded by reading titles and abstracts. We read the full text of the remaining articles. Finally, 6 studies were identified to explore the relationship between the BTLA rs1982809 polymorphism and tumor risk. Figure 1 shows the above screening process. Three studies in China, 2 in Poland, and 1 in Tunisia were conducted. CLL, RCC, ESCC, EGJA, and LC were analyzed in the study. The genotype distribution of every control group conforms to the Hardy–Weinberg equilibrium. Table 1 shows the basic information for the eligible studies.

**Table 1:** Characteristics of all included studies.

Study	Year	Country	Cancer-type	Ethnicity	Genotyping methods	Control source	Case/Control	Genotype case	Genotype control	HWE (Control)
								AA/AG/GG	AA/AG/GG	
Partyka	2016	Poland	RCC	Caucasian	TaqMan	PB	282/470	145/116/21	279/163/28	0.814
Karabon	2016	Poland	CLL	Caucasian	TaqMan	PB	321/470	156/143/22	279/163/28	0.814
Tang	2019	China	EGJA	Asian	SNPscan	HB	1205/1530	76/461/668	98/586/846	0.967
Cao	2020	China	ESCC	Asian	SNPscan	HB	713/1201	53/252/408	80/464/657	0.988
Khadhraoui	2020	Tunisia	LC	Mixed	TaqMan	PB	169/300	88/71/10	190/94/16	0.628
Wang	2021	China	LC	Asian	SNPscan	HB	988/895	71/351/566	63/361/471	0.860

The quality assessment results are shown in Table 2, where all studies had scores greater than 4, indicating that the quality of the studies included in this meta-analysis was relatively high.

**Table 2:** The NOS scores of all included studies.

Author	Year	Cancer-type	Selection				Comparability	Exposure			Score
			An adequate definition of case	Representativeness of the case	Selection of controls	Definition of controls	Control for an important factor	Assessment of exposure	The same method of ascertainment for cases and controls	Non-response rate	
–Partyka	2016	RCC	★	★	★	★	★	–	★	–	6
Karabon	2016	CLL	★	★	★	★	★	–	★	–	6
Tang	2019	EGJA	★	★	–	★	★★	–	★	–	6
Cao	2020	ESCC	★	★	–	★	★★	–	★	–	6
Khadhraoui	2020	LC	★	★	★	★	★★	–	★	–	7
Wang	2021	LC	★	★	-	★	★★	–	★	–	6

### 3.2. Meta-analysis findings

**Figure 2.**
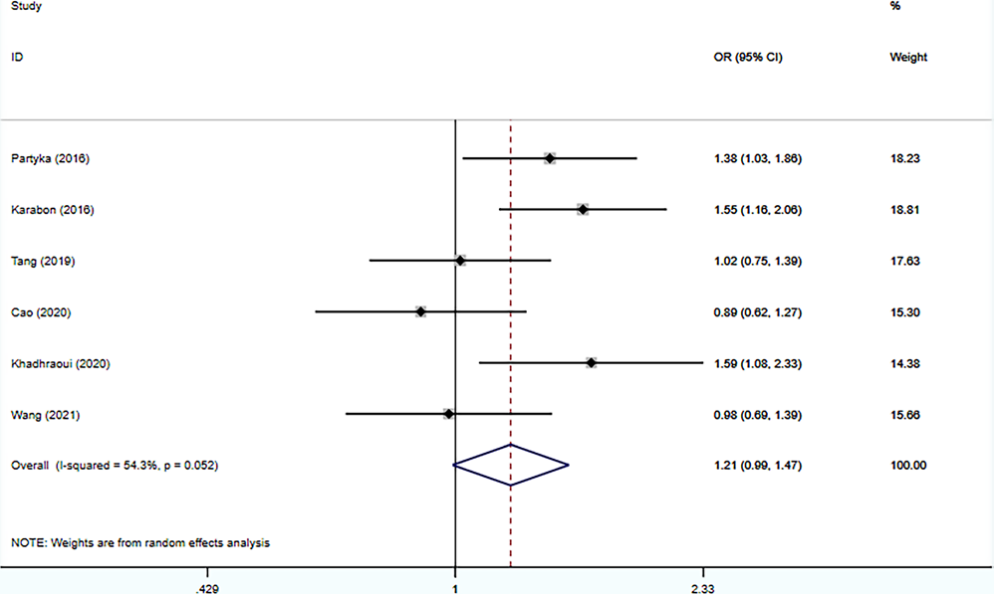
Forest plot of the dominant model (AG+GG vs AA).

**Figure 3.**
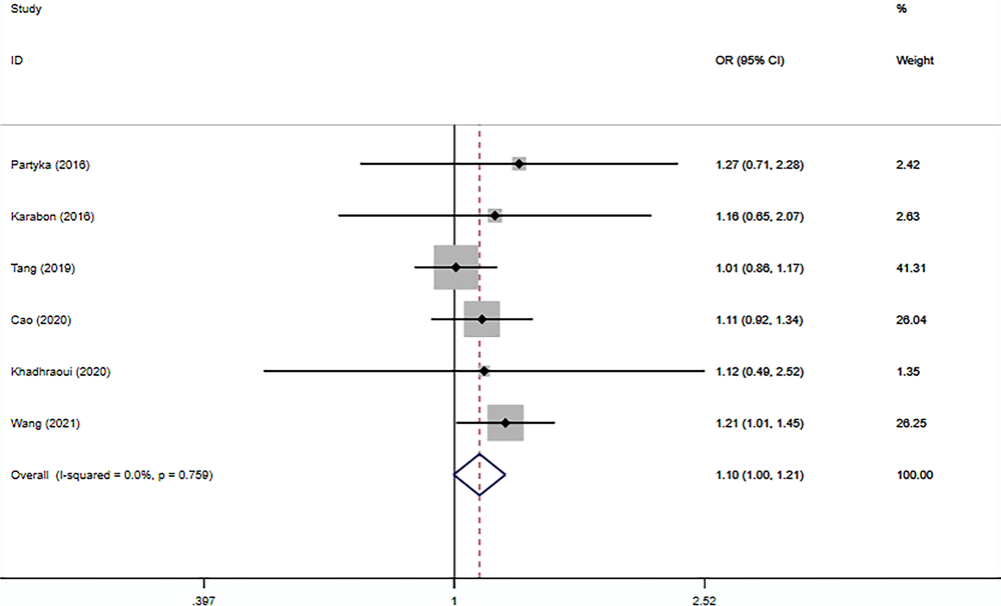
Forest plot of the recessive model (GG vs AG+AA).

**Figure 4.**
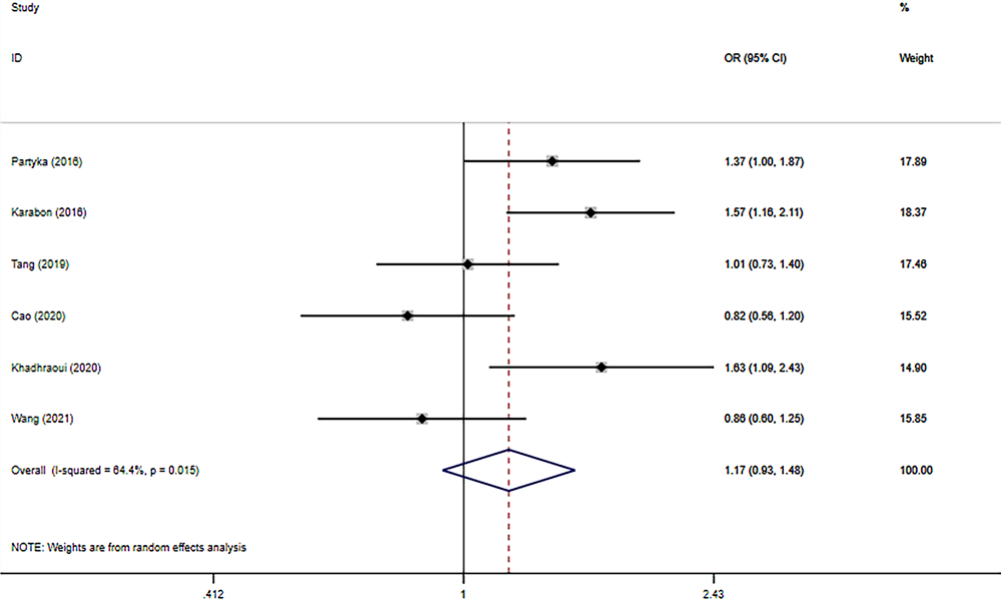
Forest plot of the heterozygous model (AG vs AA).

**Figure 5.**
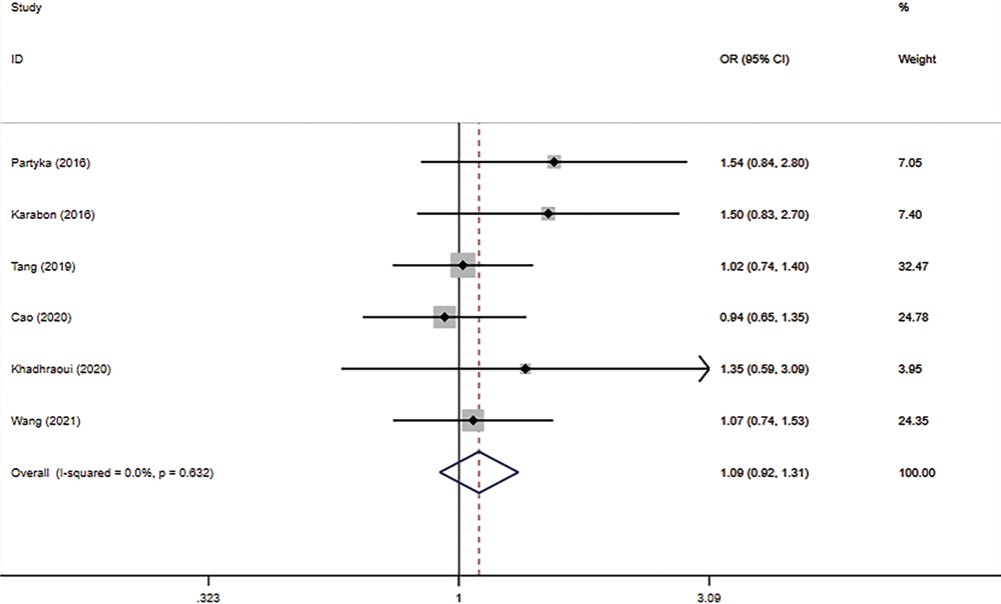
Forest plot of the homozygous model (GG vs AA).

**Figure 6.**
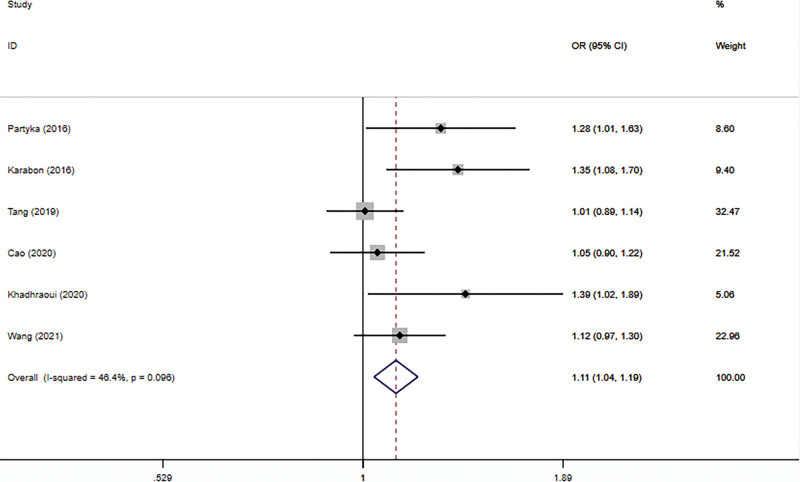
Forest plot of the additive model (G vs A).

The connection between BTLA rs1982809 polymorphism and tumor susceptibility was evaluated in 6 case-control studies, including 3678 cases and 4866 controls. And all the results can be seen in Table 3. The results revealed that rs1982809 polymorphism is a low-penetrating risk factor for cancers in the additive model (G vs. A: OR *=* 1.11, 95% CI *=* 1.04–1.19, *P*_heterogeneity_ = .096). Figures 2–6 shows Forest plots of 5 genetic models. The dominant and heterozygous models showed significant heterogeneity, so we used the random-effects model.

**Table 3:** Results of the meta-analysis.

Studies	N	Dominant model			Recessive model			Heterozygous model			Homozygous model			Additive model		
		OR (95%CI)	*P*	*I*²(%)	OR (95%CI)	*P*	*I*²(%)	OR (95%CI)	*P*	I²(%)	OR (95%CI)	*P*	*I*²(%)	OR (95%CI)	*P*	*I*²(%)
Total	6	1.21 (0.99–1.47)	.052	54.3	1.10 (1.00–1.21)	.759	0.0	1.17 (0.93–1.48)	.015	64.4	1.09 (0.92–1.31)	.632	0.0	**1.11** **(1.04–1.19**)	**.096**	**46.4**
**Ethnicity**																
Caucasian	2	**1.46** **(1.19–1.80**)	**.592**	**0.0**	1.21 (0.80–1.83)	.831	0.0	**1.47** **(1.19–1.82**)	**.536**	**0.0**	1.52 (0.99–2.31)	.951	0.0	**1.32** **(1.12–1.55**)	**.745**	**0.0**
Asian	3	0.97 (0.79–1.17)	.854	0.0	1.09 (0.99–1.20)	.313	13.8	0.91 (0.74–1.11)	.669	0.0	1.01 (0.83–1.23)	.884	0.0	1.05 (0.97–1.14)	.520	0.0
Mixed	1	**1.59** **(1.08–2.33**)	–	0.0	1.12 (0.49–2.52)	–	0.0	**1.63** **(1.09–2.43**)	.	0.0	1.35 (0.59–3.09)	–	0.0	**1.39** **(1.02–1.89**)	–	0.0
**Cancer-type**																
LC	2	1.24 (0.77–2.00)	.067	70.2	**1.20** **(1.01–1.44**)	**.854**	**0.0**	1.18 (0.63–2.20)	.022	81.0	1.11 (0.79–1.54)	.610	0.0	**1.17** **(1.02–1.33**)	**.232**	**30.1**
EN	2	0.96 (0.76–1.21)	.579	0.0	1.05 (0.93–1.18)	.432	0.0	0.93 (0.73–1.19)	.402	0.0	0.98 (0.77–1.25)	.739	0.0	1.02 (0.93–1.12)	.689	0.0
Others	2	**1.46** **(1.19–1.80**)	**.592**	**0.0**	1.21 (0.80–1.83)	.831	0.0	**1.47** **(1.19–1.82**)	**.536**	**0.0**	1.52 (0.99–2.31)	.951	0.0	**1.32** **(1.12–1.55**)	**.745**	**0.0**
**Genotyping methods**																
TaqMan	3	**1.49** **(1.24–1.79**)	**.808**	**0.0**	1.19 (0.83–1.72)	.962	0.0	**1.50** **(1.25–1.82**)	**.747**	**0.0**	**1.48** **(1.02–2.15**)	**.969**	**0.0**	**1.33** **(1.15–1.54**)	**.912**	**0.0**
SNPscan	3	0.97 (0.79–1.17)	.854	0.0	1.09 (0.99–1.20)	.313	13.8	0.91 (0.74–1.11)	.669	0.0	1.01 (0.83–1.23)	.884	0.0	1.05 (0.97–1.14)	.520	0.0
**Control source**																
PB	3	**1.49** **(1.24–1.79**)	**.808**	**0.0**	1.19 (0.83–1.72)	.962	0.0	**1.50** **(1.25–1.82**)	**.747**	**0.0**	**1.48** **(1.02–2.15**)	**.969**	**0.0**	**1.33** **(1.15–1.54**)	**.912**	**0.0**
HB	3	0.97 (0.79–1.17)	.854	0.0	1.09 (0.99–1.20)	.313	13.8	0.91 (0.74–1.11)	.669	0.0	1.01 (0.83–1.23)	.884	0.0	1.05 (0.97–1.14)	.520	0.0

Ethnicity-based subgroup analysis was performed and revealed that the risk of cancer in Caucasians was increased in 3 genetic models (AG + GG vs AA: OR *=* 1.46, 95% CI *=* 1.19–1.80, *P*_heterogeneity_ *=* .592; AG vs AA: OR *=* 1.47, 95% CI *=* 1.19–1.82, *P*_heterogeneity_ *=* .536; G vs A: OR *=* 1.32, 95% CI *=* 1.12–1.55, *P*_heterogeneity_ *=* .745). For Asians, no correlation between rs1982809 polymorphism and tumor was shown in the 5 genetic models.

Subgroup analysis based on cancer-type showed that lung cancer (GG vs AG+AA: OR *=* 1.20, 95% CI *=* 1.01–1.44, *P*_heterogeneity_ *=* .854; G vs A: OR *=* 1.17, 95% CI *=* 1.02–1.33, *P*_heterogeneity_ *=* .232) and other cancers (including RCC and CLL) were associated with rs1982809 polymorphism, but esophageal neoplasms (esophagogastric junction adenocarcinoma and esophageal squamous cell carcinoma) were not.

Different outcomes were shown in different studies using different genotyping methods. Studies using TaqMan showed rs1982809 polymorphism was associated with tumor susceptibility (AG+GG vs AA: OR *=* 1.49, 95% CI *=* 1.24–1.79, *P*_heterogeneity_ *=* .808; AG vs GG: OR *=* 1.50, 95% CI *=* 1.25–1.82, *P*_heterogeneity_ *=* .747; GG vs AA: OR *=* 1.48, 95% CI *=* 1.02–2.15, *P*_heterogeneity_ *=* .969; G vs A: OR *=* 1.33, 95% CI *=* 1.15–1.54, *P*_heterogeneity_ *=* .912); However, studies using SNPscan did not yield positive results.

As for control source, we found that rs1982809 polymorphism was associated with tumor susceptibility in the population-based studies (AG+GG vs AA: OR *=* 1.49, 95% CI *=* 1.24–1.79, *P*_heterogeneity_ *=* .808; AG vs GG: OR *=* 1.50, 95% CI *=* 1.25–1.82, *P*_heterogeneity_ *=* .747; GG vs AA: OR *=* 1.48, 95% CI *=* 1.02–2.15, *P*_heterogeneity_ *=* .969; G vs A: OR *=* 1.33, 95% CI *=* 1.15–1.54, *P*_heterogeneity_ *=* .912), but not in the hospital-based studies.

### 3.3. Sensitivity analysis

**Figure 7.**
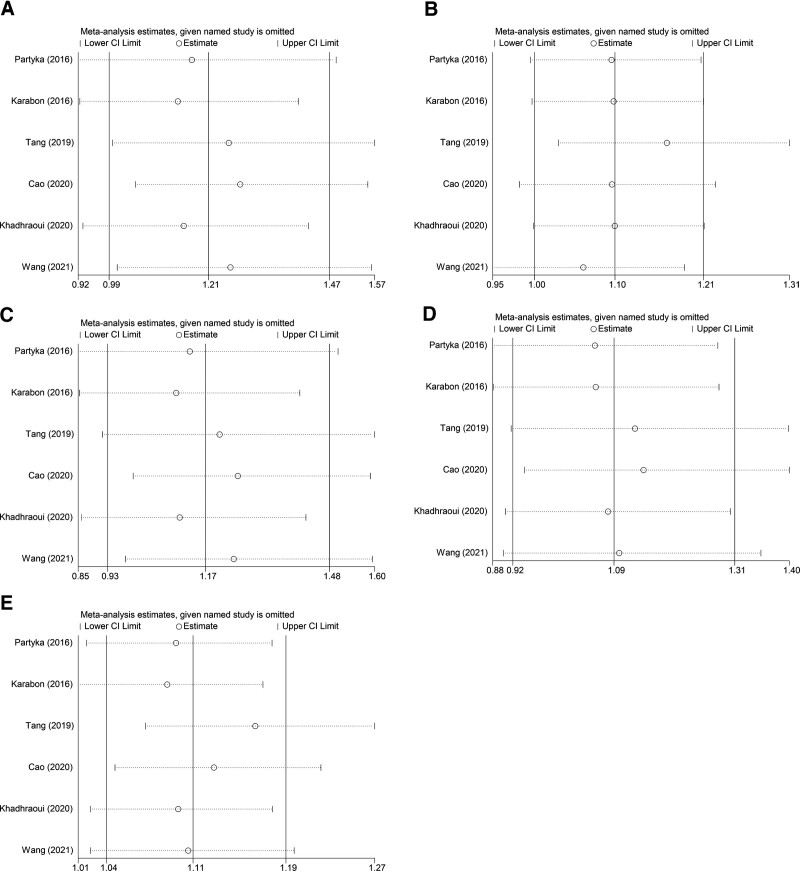
Sensitivity analysis of 5 genetic models. (A) dominant model; (B) recessive model; (C) heterozygous model; (D) homozygous model; (E) additive mode.

Sensitivity analyses were exerted in 5 models by removing 1 study at a time. Figure 7 shows the consequence of sensitivity analysis. It showed that the results of sensitivity analyses in all models suggested that the combined effects of genetic models did not change significantly, indicating that the outcomes were stabilized.

### 3.4. Publication bias

**Figure 8.**
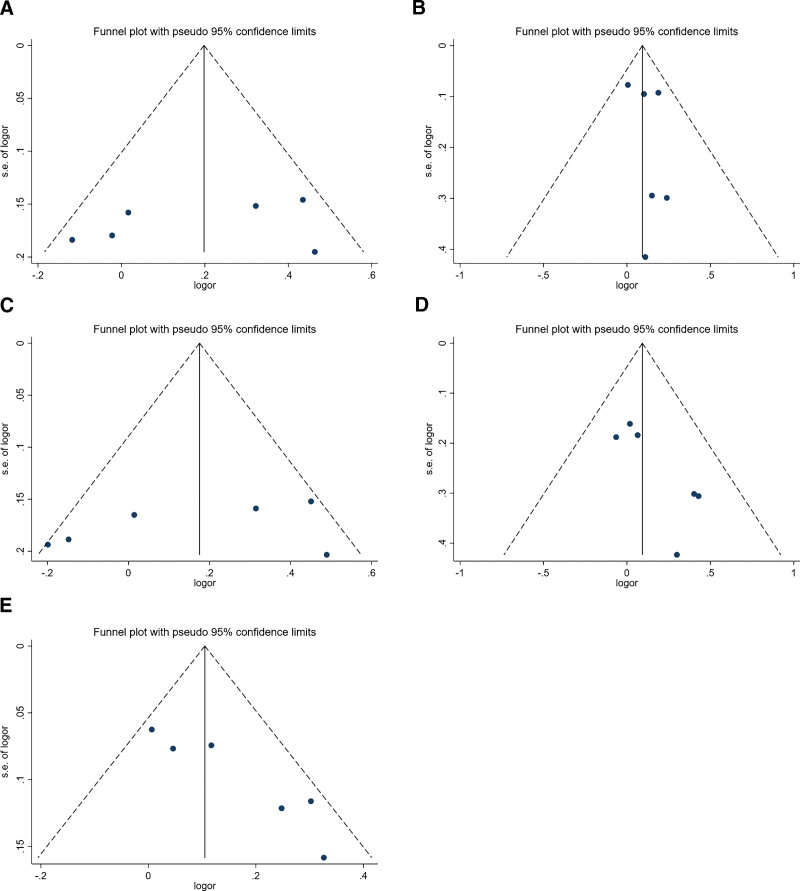
Funnel plots of 5 genetic models. (A) dominant model; (B) recessive model; (C) heterozygous model; (D) homozygous model; (E) additive mode. And the *P* values of Begg’s test are, respectively, *P*_A_ = .260, *P*_B_ = 1.000, *P*_C_ = .260, *P*_D_ = .260, and *P*_E_ = .133.

The symmetry of Begg’s funnel plots (Fig. 8) and a *P*-value of the Begg’s test in 5 models indicate that no significant publication bias in the 5 genetic models in our study.

## 4. Discussion

There is currently no meta-analysis of the relation between BTLA rs1982809 polymorphism and tumor susceptibility. Therefore, we utilized meta-analysis to explore the potential association between this SNP and the cancer risk. Compared with other models, the association between BTLA rs1982809 polymorphism and malignancy was statistically significant in the additive model.

The results of our study proved to be stable by sensitivity analysis, and there was no significant publication bias in our paper. The dominant and heterozygous models showed significant heterogeneity, so subgroup analysis was performed to explore sources of heterogeneity. Furthermore, we found that other subgroups showed no heterogeneity except tumor type, so we think the tumor type might be the source of heterogeneity.

Through subgroup analysis, we found that BTLA rs1982809 polymorphism might contribute to cancers in Caucasians, and rs1982809 polymorphism might be a risk factor for lung cancer. We found outcomes were statistically significant in the Caucasian and mixed subgroups in the ethnicity subgroup, but there was no statistical significance in the Asian subgroup. Genetic diversity, different risk factors in lifestyles, and exposure to different environmental factors might bring out the differences. Moreover, it may also have something to do with the lack of data. In the tumor type subgroup, LC is associated with rs1982809 polymorphism. And conspicuous heterogeneity was found in the subgroup of LC; the reasons may be ethnicity and genotyping methods because 2 studies of the LC group used different genotyping methods and included participants from different ethnic groups.

BTLA will take a particular place in immunotherapy for tumors according to robust evidence offered by a sufficient number of studies. The overexpression of BTLA and HVEM is concerned with the progression and adverse outcomes of gastric cancer by Lan X et al., and BTLA/HVEM pathway is deemed to be a potential treatment option for gastric cancer.^[23]^ Li et al. reported that high BTLA expression might portend a poor prognosis for patients with Non–Small-Cell Lung Cancer, as well as represent a new immunotherapy target.^[24]^ BTLA observed in carcinoma tissue can predict poor outcomes of patients with epithelial ovarian carcinoma (EOC) in the study of Chen et al., and potential clinical value showed in the combined application of chemotherapy and anti-BTLA antibody for the therapy of EOC patients.^[25]^ Liu et al. found that BTLA/HVEM might serve as an attractive target for hepatocellular carcinoma immunotherapy.^[26]^ However, some research showed diverse outcomes, a study showed that BTLA is an underlying factor for prolonged survival in colorectal cancer (CRC).^[27]^ To sum up, BTLA may be a new and powerful immunotherapy target after PD-1 and CTLA-4. Therefore, studies of BTLA gene polymorphism are of great significance to further prove that BTLA is a potential target for immunotherapy.

However, our research also has some limitations. First, the lack of data in this study led to an inability to analyze some clinical factors, including age, gender, living conditions, and biochemical characteristics. Second, the sub-group analysis did not include the black human race.

All in all, our study is significantly meaningful, we found that rs1982809 polymorphism in BTLA may be a risk factor for cancer, especially in Caucasians, and this SNP might contribute to lung cancer. However, well-designed studies with larger sample sizes and multicenter are required to further probe the connection between the SNP of rs1982809 and cancer susceptibility.

## Author contributions

Study design/planning: Yingliang Li, Jian Chen.

Data collection/entry: Jian Chen, Jun Wang, Ruihao Liu.

Data analysis/statistics: Jian Chen, Xuan Liu, Mingjun Shang.

Data interpretation: Yingliang Li, Jian Chen, Qiang Li.

Preparation of manuscript: Yingliang Li, Jian Chen, Yingying Liu, Mingzhi Zha.

Literature analysis/search: Jian Chen, Jun Wang, Ruihao Liu.

Founds collection: Yingliang Li.
